# Decline of the Ghost Orchid, *Dendrophylax lindenii*, in Florida and Cuba as Revealed by Temporal Dynamic Studies Spanning the Past Decade

**DOI:** 10.3390/plants15213483

**Published:** 2026-11-07

**Authors:** Adam R. Herdman, Ernesto B. Mújica, Lawrence W. Zettler, Elaine H. González, Alejandro Camejo Vergara, Mark W. Danaher, Michael E. Kane, Héctor E. Pérez, Carrie Reinhardt Adams

**Affiliations:** 1 Horticultural Sciences Department, University of Florida, Gainesville, FL 32611, USA; micropro@ufl.edu (M.E.K.); heperez@ufl.edu (H.E.P.); rein0050@ufl.edu (C.R.A.); 2 Orquideario Soroa, QXVR+2MM Carretera a Soroa Km. 8, Candelaria 22700, Cuba; ernestomujica2760@gmail.com (E.B.M.);; 3 Km. 2 ½ Carretera a Luis Lazo, Pinar del Río 20100, Cuba; 4 Department of Biology, Illinois College, 1101 W College Avenue, Jacksonville, IL 62650, USA; lwzettle@ic.edu; 5 Guanahacabibes National Park, Carretera a La Bajada, Sandino, Pinar del Río 24150, Cuba; alejandrocamejo83@gmail.com; 6 Florida Panther National Wildlife Refuge, U.S. Fish & Wildlife Service, Immokalee, FL 34142, USA; mark_danaher@fws.gov

**Keywords:** plant demography, conservation biology, reproductive output, epiphytic orchids, population viability, long-term monitoring, hurricane disturbance

## Abstract

The ghost orchid, *Dendrophylax lindenii*, is a rare leafless epiphyte restricted primarily to southern Florida and western Cuba. Despite a decade of conservation efforts, the understanding of in situ long-term stressors remain understudied. Building on previous demographic studies, we extended annual monitoring of *D. lindenii* at the Florida Panther National Wildlife Refuge from 2015 to 2024 and at Guanahacabibes National Park, Cuba, from 2006 to 2023, to provide a direct long-term comparison of population trajectories across two major portions of the species range. We evaluated total trends and regional differences, changes in demographic size structure, and associations between annual population growth and regional climate conditions and disturbance. Both populations declined through time, but the decline was greater in Florida, where totals decreased by 8.8% every year from a maximum of 529 individuals in 2017 to 237 in 2024, compared with a decline of 2.5% from 360 individuals in 2006 to 195 in 2023 in Cuba. Plants were grouped by mean living root length C1 (0–8.0 cm), C2 (8.1–11.3 cm), C3 (11.31–15.3 cm), and C4 (>15.3 cm). In Florida, C1 and C3 declined significantly, while losses of large C4 plants also occurred. In Cuba, C1, C2, and C3 declined while C4 increased, retaining a greater number of large reproducing plants. Flowering fluctuated in Florida but increased in Cuba, while fruit production remained low in both regions. Regional temperatures increased during the study period, but annual population size was not statistically associated with temperature, precipitation, or tropical cyclone occurrence. Class-based projections indicated continued declines in both regions, with a stronger projected decrease in Florida. These declines indicate that conservation actions should be tailored to local population structure. Protection of existing large reproductive plants, supplementation of depleted smaller-size classes, and coordinated management across remaining populations may provide the strongest basis for sustaining *D. lindenii* in Florida and Cuba.

## 1. Introduction

*Dendrophylax lindenii* (Lindley) Bentham ex Rolfe (Angraecinae, Orchidaceae) is a rare epiphytic orchid restricted primarily to southern Florida and western Cuba. Small population sizes, limited distribution, and co-habiting relationships make quantitative population assessment integral for its conservation [1,2]. Additional stressors compound these sensitivities, such as illegal collection due to its horticultural value and difficulty in cultivation [3]. For rare species, repeated demographic surveys are necessary to determine whether short-term fluctuations represent persistent stressors. This is especially important for epiphytic orchids, for which long-term demographic studies remain uncommon, largely because many sites are often difficult to access [4,5].

**Figure 1:**
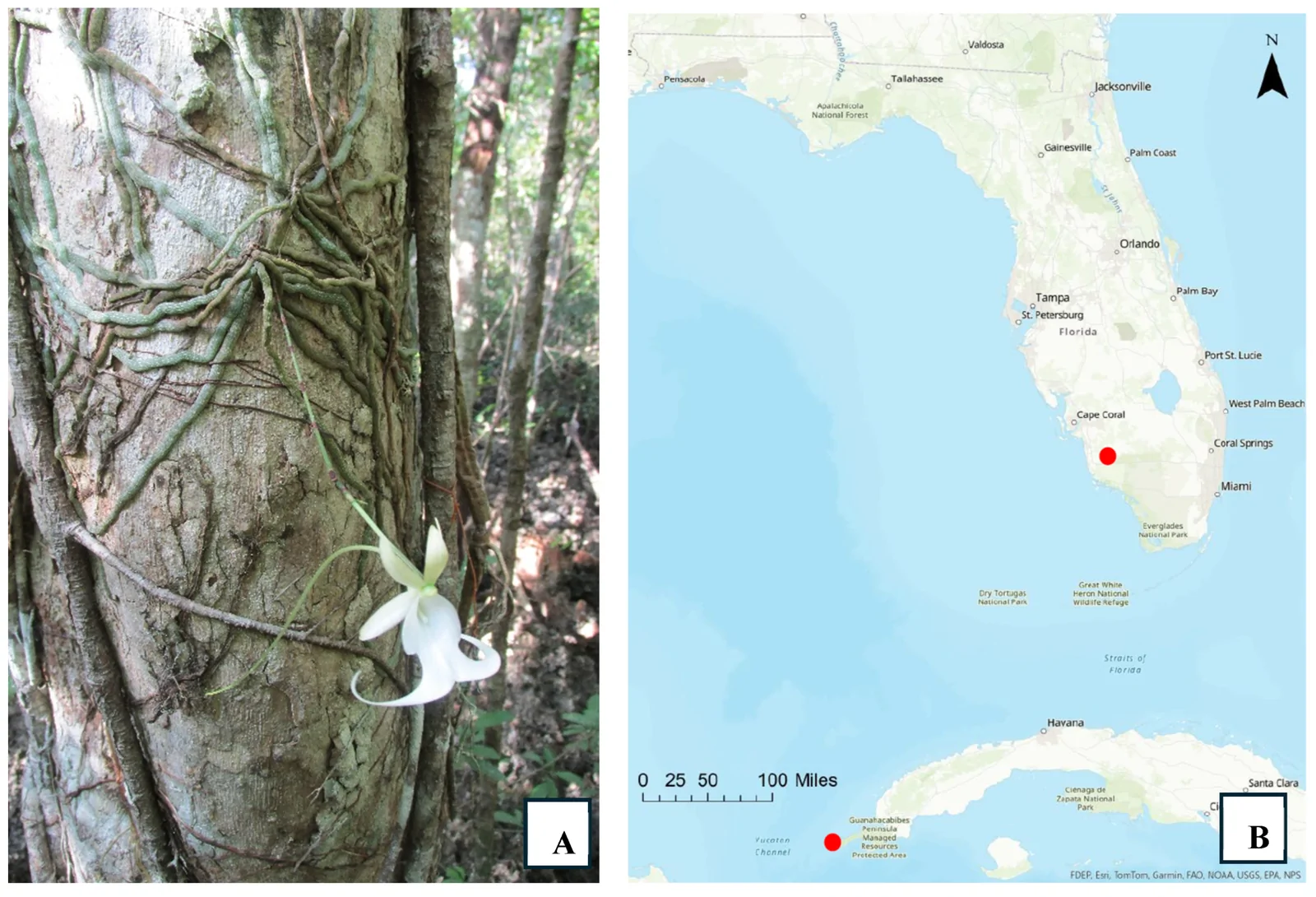
*D. lindenii* (**A**) in flower and (**B**) locations in southern Florida and western Cuba, where major extant populations of *D. lindenii* occur at the FPNWR and Guanahacabibes National Park study regions. Photograph: L.W. Zettler.

*Dendrophylax lindenii* occurs in contrasting habitats across its range as a leafless epiphyte (Figure 1). The species develops from a protocorm into a leafless seedling in which roots become the dominant vegetative and photosynthetic organs; new roots arise from the reduced shoot apex beneath older roots [6]. In Florida, populations are concentrated in the seasonally flooded forests of the Big Cypress Basin (Figure 1B), where plants occur primarily on pop ash (*Fraxinus caroliniana*) and pond apple (*Annona glabra*) [7,8,9]. In western Cuba, the species occurs in semi-deciduous forests on a broader assemblage of host trees [7,10]. Most known populations occur in southern Florida and Guanahacabibes National Park, although limited historical records exist elsewhere in Cuba [11]. Additional locations such as the Bahamas remain unconfirmed [12,13]. These regional differences provide an opportunity to compare population dynamics of the same species under contrasting environmental conditions.

Orchid population dynamics reflect interacting biological and environmental processes affecting survival, reproduction, and recruitment, including pollination, mycorrhizal associations, competition, microsite conditions, and climate [6,14,15,16,17,18,19,20,21]. Because these processes vary among sites and years, changes in observed population counts alone cannot identify the mechanisms responsible. Long-term monitoring of individual counts and demographic size structure is therefore important for distinguishing persistent population trends from short-term fluctuations and for identifying potential demographic bottlenecks relevant to conservation management.

For epiphytic orchids, the distribution of suitable host trees is one component of habitat availability, but host-tree presence alone does not ensure successful establishment. Tree-associated microsites vary in moisture conditions, bark characteristics, and associations with compatible mycorrhizal fungi [6,7,16,17]. In southern Florida, historical logging, land-use changes, and altered hydrology have modified habitats supporting *D. lindenii* [22]. Habitat degradation can take several forms, including wetland drainage, forest fragmentation, and increased exposure to habitat edges. Fragmentation may reduce habitat connectivity and alter local microclimatic conditions and ecological interactions [23]. Studies of other rare plants in southern Florida have also demonstrated associations between habitat fragmentation, disturbance history, and plant abundance [24]. Because *D. lindenii* depends on host trees, mycorrhizal fungi, and pollinators, these changes could affect multiple ecological processes important for orchid establishment and persistence [6,7,14,15,16,17]. Genetic diversity and gene flow could also influence population responses to environmental change, although their contributions to the long-term demographic trends of *D. lindenii* remain untested. These ecological and genetic factors provide context for the contrast between Florida and Cuba, but the present monitoring records do not distinguish their individual effects.

Despite substantial research on *D. lindenii*, long-term demographic information remains limited. This is a broader problem in epiphyte ecology, where long-term population and community datasets remain rare [5]. Epiphytic population growth can vary according to microsites, making short monitoring periods difficult to interpret [25]. Studies in Cuba documented prolonged demographic effects following hurricane disturbance [26,27], while an initial six-year assessment at the Florida Panther National Wildlife Refuge (FPNWR) projected continued decline and insufficient recruitment to offset losses [28]. Whether these trends persisted with additional monitoring, and whether comparable size-class trajectories occurred in Cuba and Florida, remained unresolved.

Long-term environmental changes further reinforce the need for long-term monitoring of rare species. Environmental conditions vary over temporal scales that exceed most monitoring studies, which can make it difficult to interpret short-term datasets. Periodic disturbances may be a natural component of an ecosystem, yet their impacts on a population’s demography may only become apparent when followed across multiple disturbance and recovery periods. Tropical cyclones can damage orchids, host trees, and forest canopy structure [26,27,29,30,31,32]. Studies in Guanahacabibes showed that *D. lindenii* experienced reduced population growth following Hurricane Ivan and incomplete recovery during subsequent years [26,27]. Similar hurricane impacts have been reported for other tropical epiphytes [33,34]. These responses show that disturbance effects may persist beyond the year in which a storm occurs.

To combat the challenge of research and management of a species with complex ecology, structured modeling is an important tool for interpreting long-term demographic datasets and identifying the processes contributing to observed changes over time [18,20]. Matrix population models and integral projection models can identify how survival, growth, reproduction, and transitions among size classes contribute to population growth, while sensitivity and elasticity analyses help determine which demographic processes most strongly influence population persistence [18,21]. These approaches can also reveal demographic bottlenecks, such as insufficient recruitment or loss of large reproductive individuals [19,21]. Identifying these bottlenecks is particularly important for rare orchids, where limited recruitment or the loss of established reproductive individuals may constrain population recovery.

Niche modeling has suggested that climatic changes may alter suitable conditions for *D. lindenii* and associated species differently in Florida and Cuba [35]. Alteration of local conditions also affects epiphyte distribution and community structure [34,36,37]. Together, development and environmental changes contribute to shifting distribution over time, with several tropical plant and animal taxa expanding or establishing northward from the Caribbean [38,39,40]. In Florida, *D. lindenii* recruitment has previously been concentrated in monitored populations occurring in wettest locations [28]. However, broad environmental associations do not by themselves identify the mechanisms responsible for population change. Long-term demographic records are therefore needed to establish population trajectories before potential drivers can be evaluated.

Beginning in 2015, coordinated monitoring in Florida and Cuba provided an opportunity to compare long-term demographic trajectories of *D. lindenii* across two major portions of its range. The populations occur under contrasting environmental and ecological regimes, allowing evaluation of whether similar demographic patterns emerge across distinct regional settings.

Here, we compare annual monitoring records from the FPNWR from 2015 to 2024 and Guanahacabibes National Park, Cuba, from 2006 to 2023. We evaluated (1) temporal population trends and regional differences, (2) changes in population demographic size structure, and (3) regional climate and disturbance associations with population size. Together, these datasets provide a long-term comparison of *D. lindenii* population trajectories across two major portions of its range.

## 2. Results

### 2.1. Observed Populations, Demographic Structure, Climate and Disturbance

**Figure 2:**
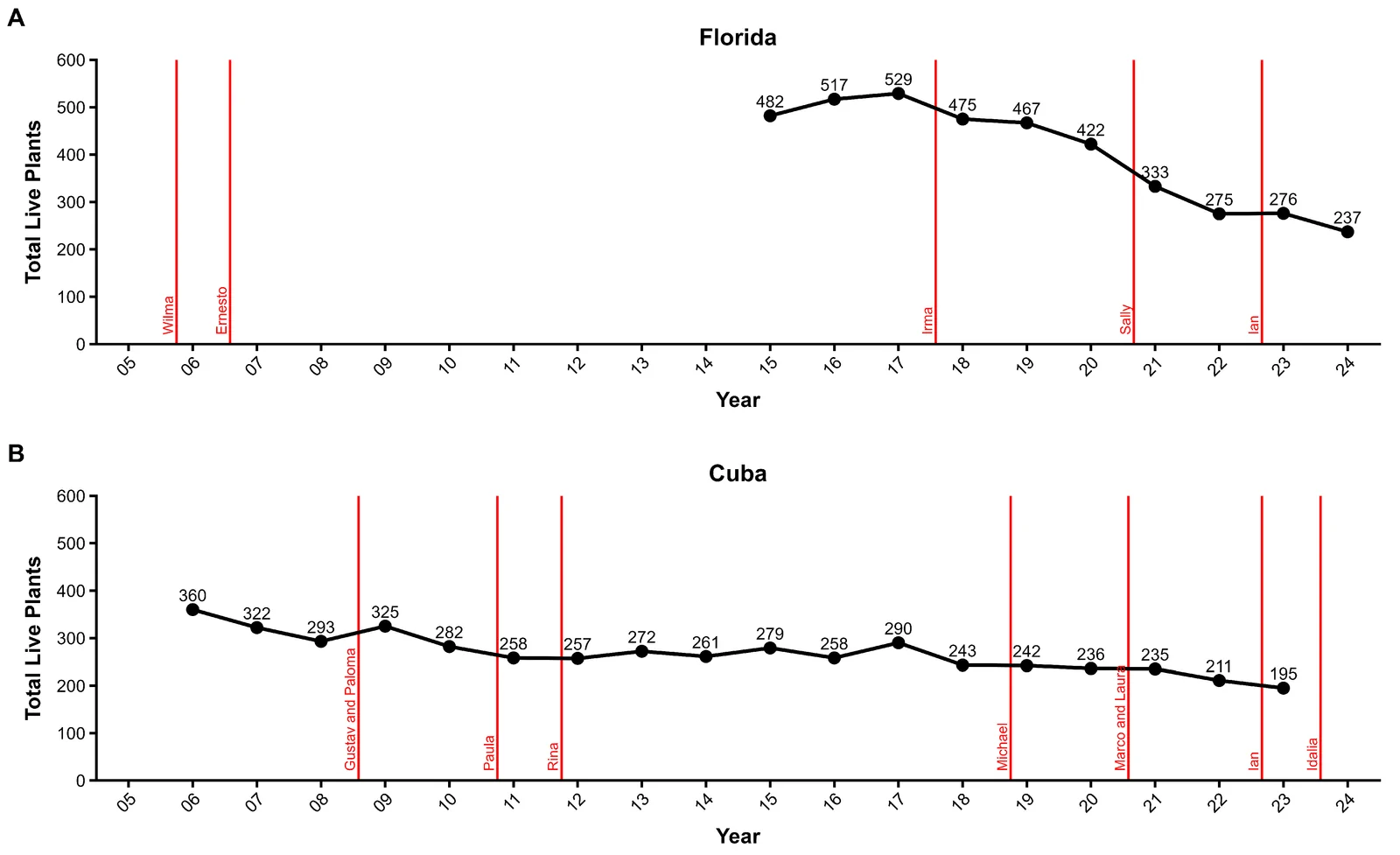
Annual counts of living *Dendrophylax lindenii* individuals monitored and timing of tropical cyclones in (**A**) Florida in the FPNWR from 2015 to 2024 and (**B**) Cuba in Guanahacabibes National Park, Cuba, from 2006 to 2023.

Long-term surveys revealed a decline in the number of live *D. lindenii* in Florida and Cuba. In Florida, the number of live plants increased from 482 individuals in 2015 to a maximum of 529 individuals in 2017, followed by a sustained decline to 237 individuals by 2024 (Figure 2A). The estimated annual decline was 8.8% (Table 1). In Cuba, total population declined from 360 live plants in 2006 to 195 in 2023 (Figure 2B), corresponding to an annual decline of 2.5% (Table 1). The rate of decline differed between regions, with a greater decline in Florida than in Cuba (population by year interaction, *p* = 0.0189).

**Figure 3:**
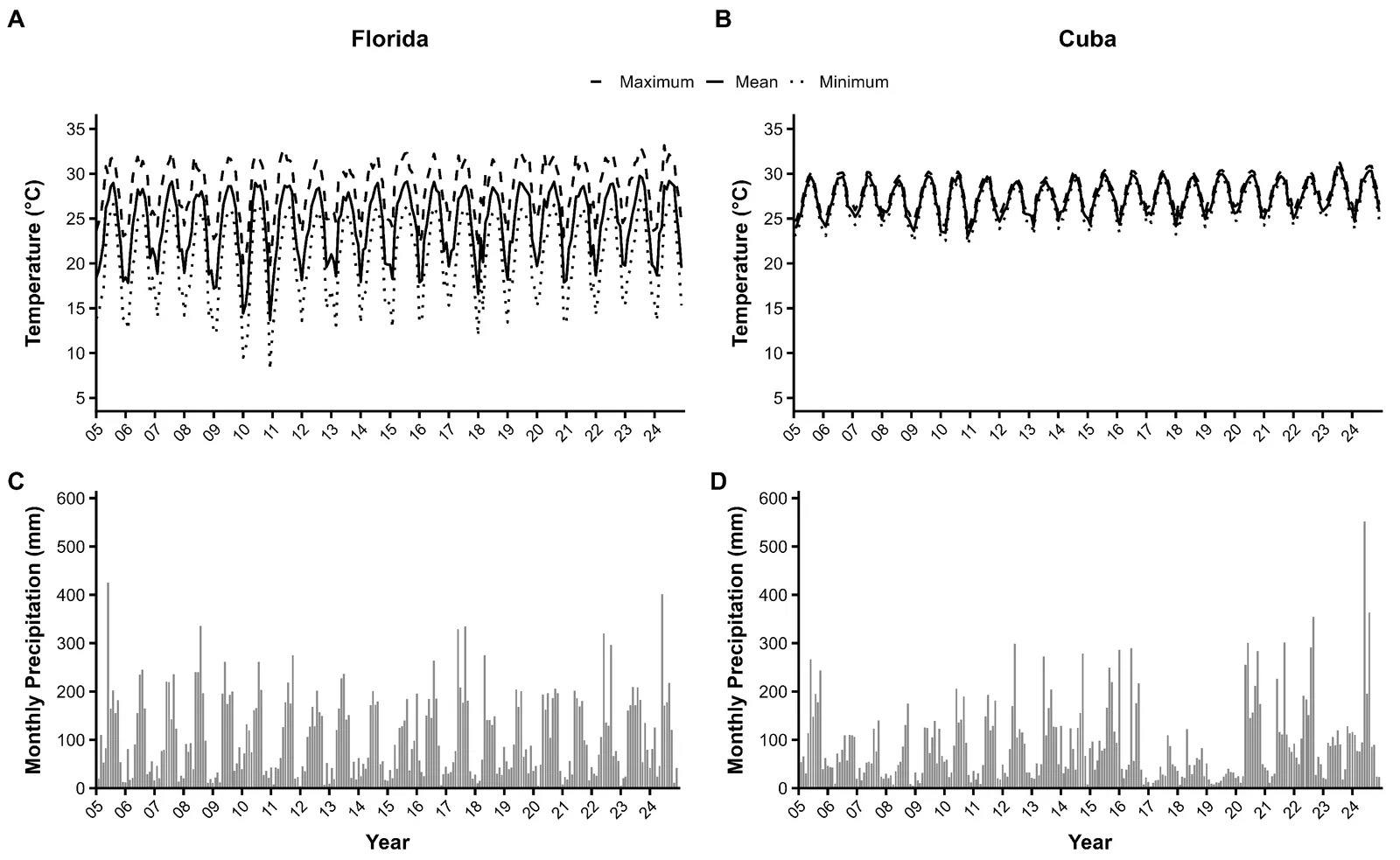
Monthly temperature and precipitation patterns for the Florida and Cuba study regions from 2005 through 2024. Monthly maximum, mean, and minimum temperature for the FPNWR (**A**,**C**), and Guanahacabibes National Park region in Cuba (**B**,**D**). Temperature and precipitation data were obtained from NASA POWER and are presented to illustrate regional climatic variation during the monitoring period. Climate data were obtained from the National Aeronautics and Space Administration (NASA) Prediction Of Worldwide Energy Resources (POWER) project. Daily POWER Data v10 were accessed on 31 August 2026.

Regional climate records showed increased warming during 2005–2024 (Figure 3). Mean temperature increased by approximately 1.47 °C in Florida (95% CI: 0.95–1.98 °C) and 1.05 °C in Cuba (95% CI: 0.78–1.32 °C), with increases also detected in minimum and maximum temperatures. No consistent directional precipitation trend was detected in Florida, while precipitation in Cuba varied nonlinearly through time without a considerable net change between the beginning and end of the study period.

Although both regions experienced measurable environmental changes during the study period, the regional climate variables examined did not correspond with the year-to-year population changes observed for *D. lindenii*. Linear regressions found no evidence that annual temperature or precipitation variables explained variation in population trends for either Florida or Cuba. Spearman’s rank correlations likewise did not detect associations between annual population trends and temperature or precipitation in the same year, and one-year-lagged correlations also showed no consistent relationships. Permutation tests also found no difference in annual population growth disturbance intervals in either Florida or Cuba. These results indicate that the broad climate and disturbance variables evaluated here did not account for the observed demographic declines over the monitoring periods.

**Figure 4:**
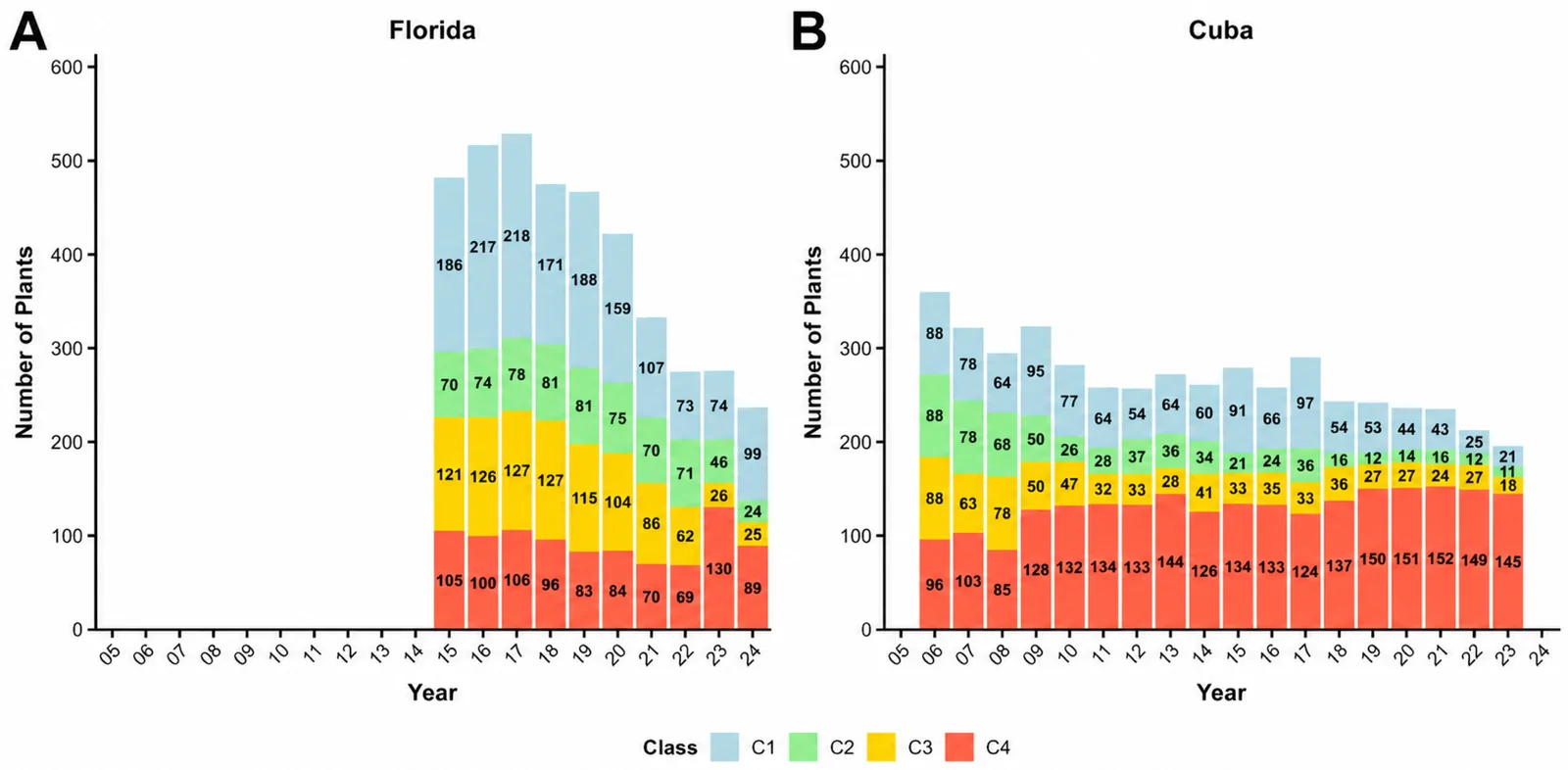
Annual demographic size-class structure of *D. lindenii* populations in (**A**) Florida and (**B**) Cuba. Stacked bars show the number of observed individuals in each of four classes based on mean living root length: C1 (0–8.0 cm), C2 (8.1–11.3 cm), C3 (11.31–15.3 cm), and C4 (>15.3 cm).

Temporal trends among demographic size classes differed between Florida and Cuba (Table 1; Figure 4). In Florida, C1 numbers declined through time at an estimated rate of 11.5% per year, while C3 declined at a rate of 16.7% per year. In contrast, no evidence of a temporal trend was detected for C2 or C4 after correction for multiple comparisons (Table 1).

In Cuba, C1, C2, and C3 declined gradually through time, with estimated annual declines of 5.6%, 10.6%, and 6.5%, respectively (Table 1). In contrast, the number of C4 plants increased by an estimated 2.4% per year. This contrasting pattern resulted in a pronounced change in Cuban *D. lindenii* size structure, with C4 increasing from approximately 27% of classified individuals in 2006 to 74% in 2023. Thus, the overall population decline occurred despite increasing C4 demographic and was associated primarily with losses among C1, C2, and C3.

*Dendrophylax lindenii* structure differed between Florida and Cuba, particularly in the relative number of C4 individuals. In Cuba, C4 plants (individuals with a mean root length ≥ 15.3 cm) represented more than half of the population in every survey year, ranging from approximately 51% in 2006 to 84% in 2023. The proportion of C4 individuals also increased through time, especially after 2018, and so the Cuban population was increasingly dominated by larger mature plants (Figure 4). In contrast, C4 individuals represented a much smaller proportion of the Florida population, ranging from approximately 17.5% to 25% between 2015 and 2022. Florida populations were instead more evenly distributed among C1, C3, and C4 classes, although all three classes declined after 2017.

**Figure 5:**
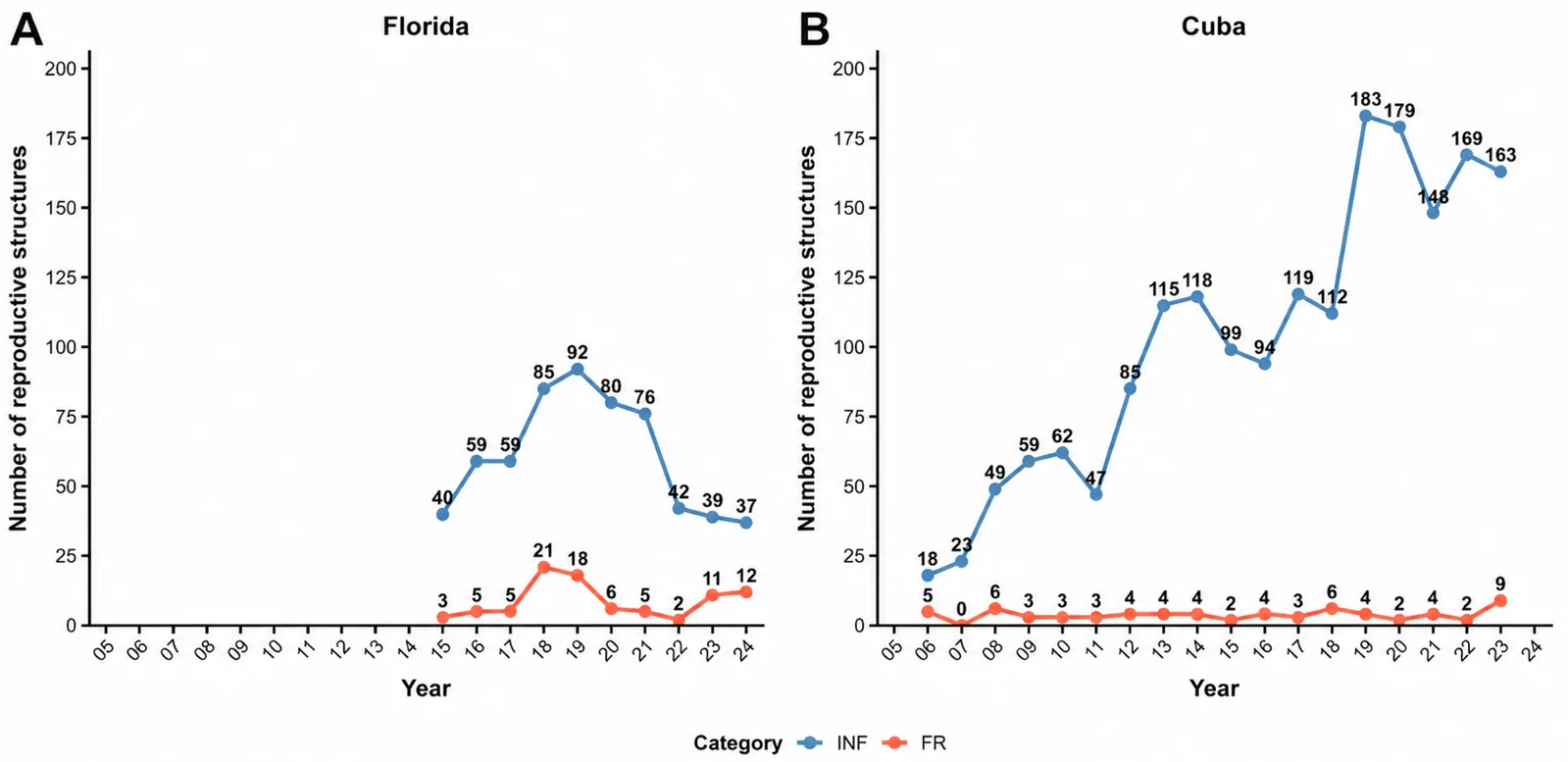
Annual reproductive output of. *D. lindenii* populations in Florida (**A**) and Cuba (**B**). Lines show the number of observed inflorescences (INF) and fruits (FR) recorded during annual surveys.

Reproductive output differed between Florida and Cuba in regard to reproductive structures. In Florida, *D. lindenii* inflorescence production increased from 40 flowers in 2015 to a peak of 92 in 2018, then declined to 37 by 2024 (Figure 5). Fruit production remained low relative to inflorescence production, peaking at 21 fruits in 2018 before declining to two fruits by 2022. When standardized by total live plants, inflorescences represented 8.3–22.8% of the Florida population, whereas fruits represented only 0.6–5.1%.

In Cuba, inflorescence production increased more consistently through time, rising from 18 inflorescences in 2006 to 183 in 2018 and remaining relatively high through 2023 (Figure 5). Inflorescences represented 5.0–83.6% of total live plants across the Cuban survey period and exceeded 50% of total live plants in several recent years. Despite this high proportional inflorescence production, fruit production remained consistently low, ranging from 0 to nine fruits annually, or 0–4.6% of total live plants. Thus, both populations exhibited low fruit production relative to inflorescence production, but Cuba consistently showed much higher proportional flowering than Florida.

### 2.2. Simplified Population Trajectories and Projected Class Dynamics

**Figure 6:**
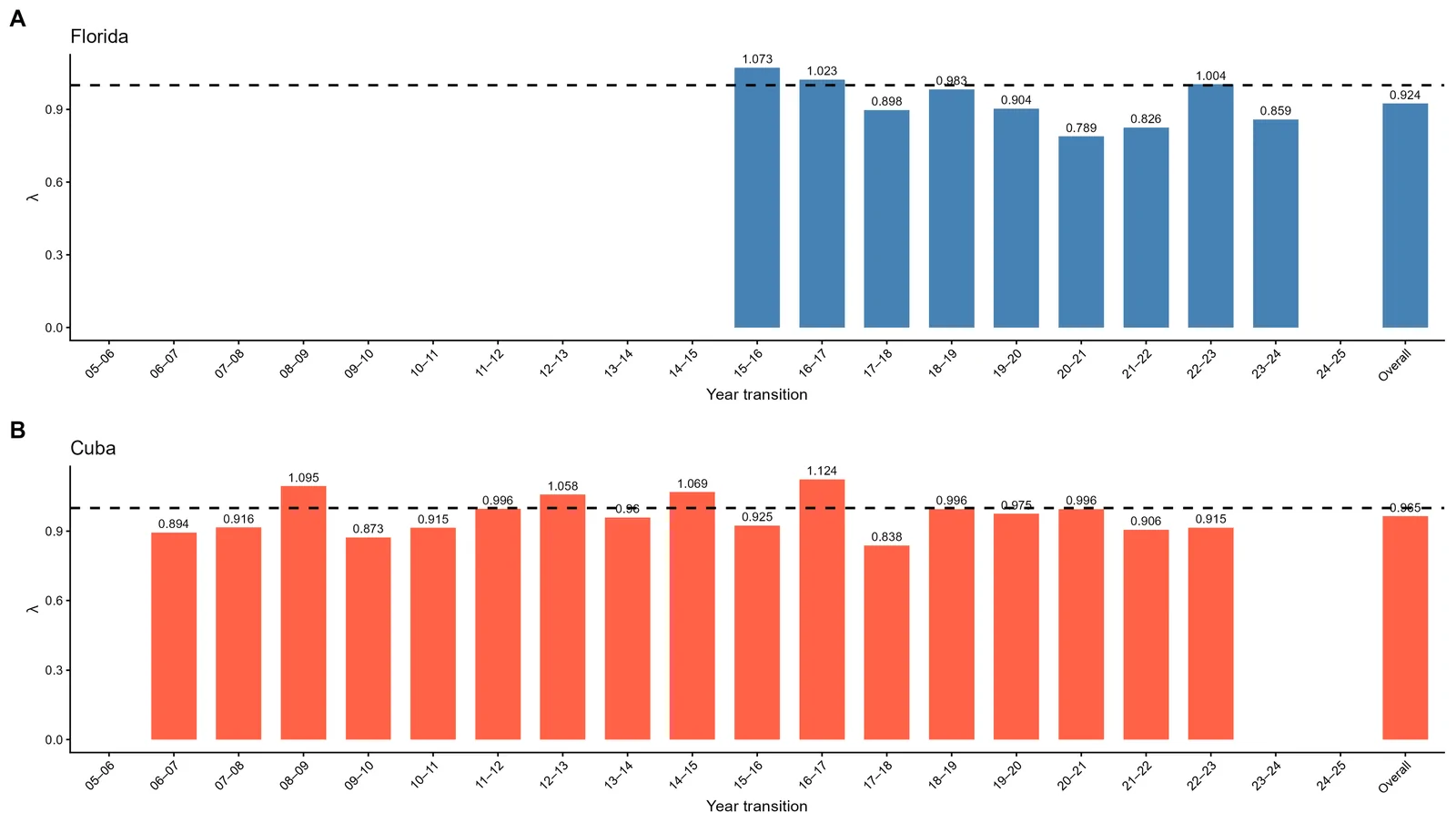
Annual finite rates of apparent population change (λ) for *D. lindenii* populations in Florida (**A**) and Cuba (**B**), calculated from year-to-year changes in aggregate class-structured number of plants. Bars represent λ for each annual transition, and the dashed horizontal line indicates λ = 1.

Annual finite rates of population change (λ) calculated from successive aggregate class counts indicated overall declining population trajectories in both regions, although year-to-year patterns differed between Florida and Cuba. In Florida, λ exceeded 1 during the 2015–2016, 2016–2017, and 2022–2023 transitions, and was below 1 during the remaining annual transitions. The lowest annual value occurred during the 2020–2021 transition, when λ declined to 0.789. The overall geometric mean λ for Florida was 0.924 (95% bootstrap CI: 0.868–0.986), providing additional support for an overall declining population trajectory across the study period (Figure 6). These values describe changes in recorded class-structured count rather than directly measured individual survival or recruitment rates.

In Cuba, annual λ values fluctuated more frequently around 1.0. Values exceeded 1 during the 2008–2009, 2012–2013, 2014–2015, and 2016–2017 transitions, with the highest annual value occurring from 2016 to 2017 (λ = 1.124). However, most annual transitions were below 1, and the overall geometric mean λ for Cuba was 0.965 (95% bootstrap CI: 0.927–1.004). Although the point estimate was below 1, the confidence interval overlapped with population stability, reflecting greater interannual variation in population change. Over time, both populations declined, but the Florida population exhibited a stronger and more consistent recent decline, whereas the Cuban population showed a slower decline with intermittent years of population increase.

**Figure 7:**
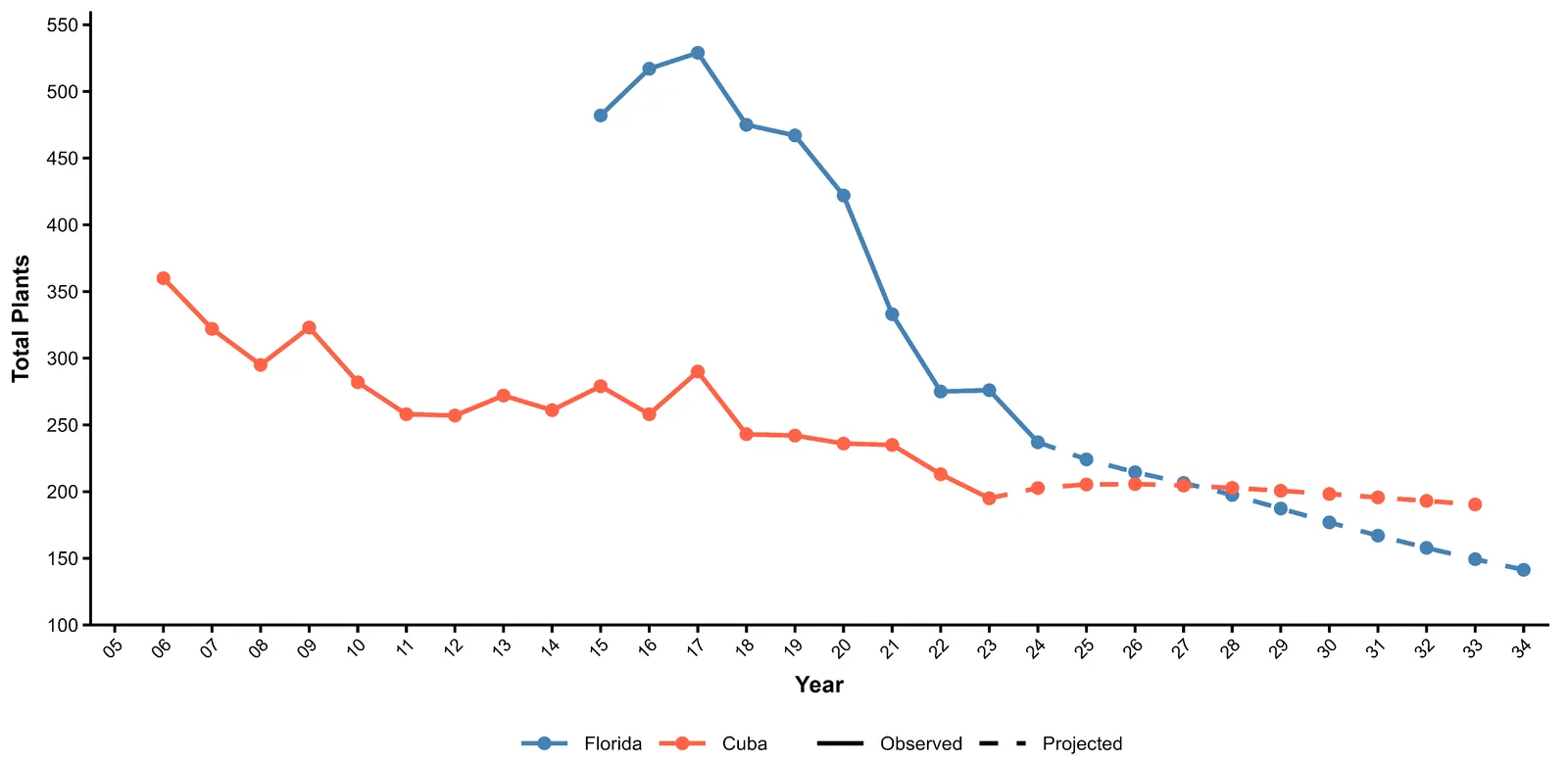
Observed and projected total populations of *D. lindenii* in Florida and Cuba within evaluated sites. Solid lines represent observed annual counts of total plants, and dashed lines represent projected population trajectories derived from simplified class-based demographic modeling using aggregate annual counts. Florida is shown in blue and Cuba in red. Projected values illustrate the continuation of fitted demographic patterns and do not represent independently validated estimates of extinction probability or timing.

Projections based on the overall count data also indicated continued population decline in both regions. Observed Florida numbers in *D. linenii* declined sharply after 2017, and projected values continued to decrease over the modeled period (Figure 7). Cuban totals declined more gradually during the observed survey period, and projections suggested a slower continued decline. Overall, the projected total number of plants indicated a steeper decline in Florida than in Cuba. The dominant eigenvalue from the aggregate stage model was 0.946 for Florida (95% bootstrap CI: 0.854–1.022) and 0.985 for Cuba (95% CI: 0.952–1.015). Both confidence intervals included 1.0, indicating these model-derived growth rates were associated with substantial uncertainty and were interpreted as descriptors of apparent class-structured trajectories rather than definitive estimates of population growth.

These projections were generated from aggregate annual class counts rather than individual plant histories, illustrating population trajectories conditional on the fitted class-level relationships, and should not be interpreted as independently validated predictions of population persistence or the timing of local extirpation.

**Figure 8:**
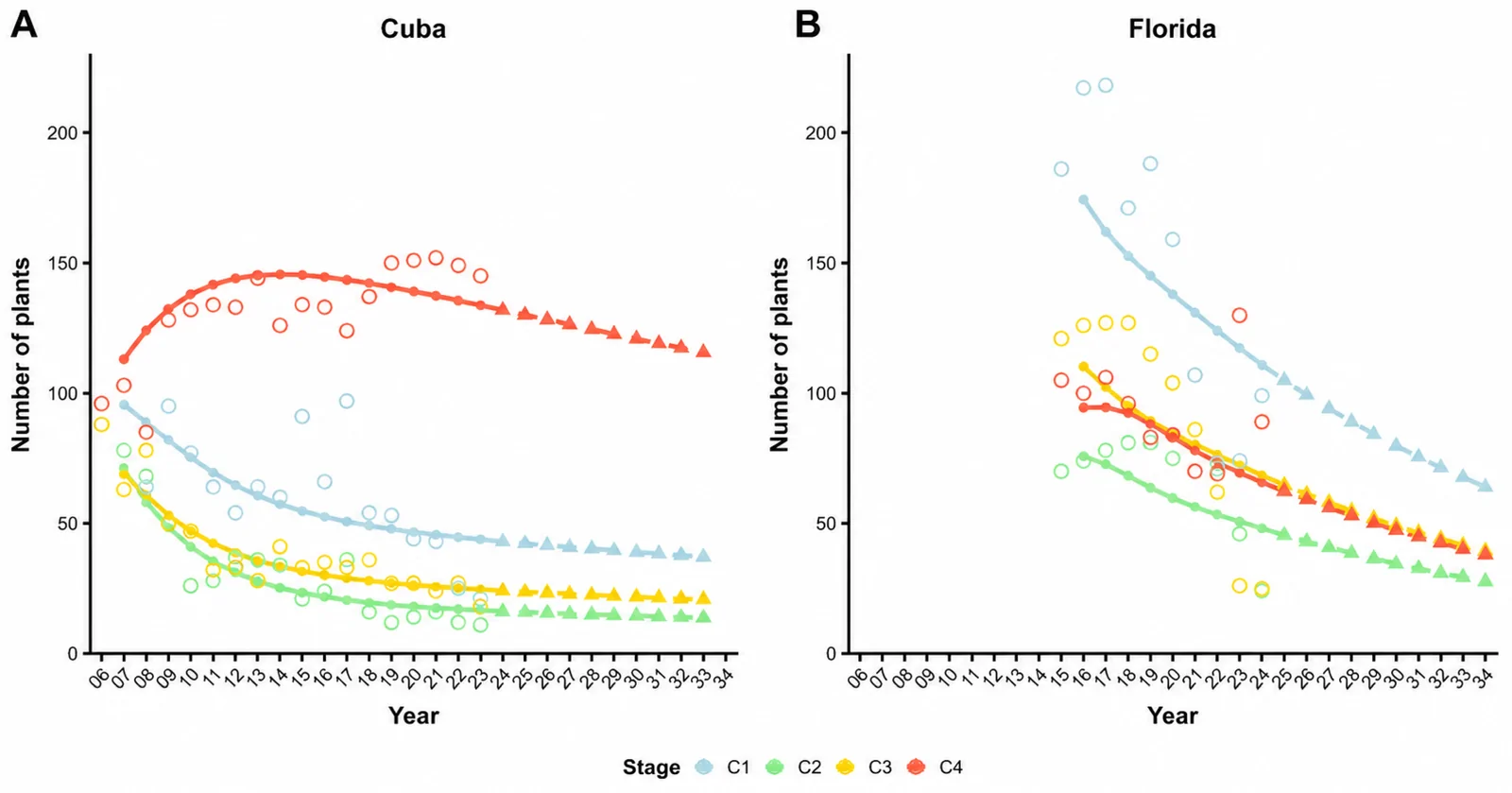
Observed, modeled, and projected size-class number of *D. lindenii* plants in Cuba (**A**) and Florida (**B**). Open circles represent observed annual class counts, solid-colored lines with circles represent modeled values during the observed survey period, and dashed lines with triangles represent projected trajectories beyond the observation period. C1 (0–8.0 cm), C2 (8.1–11.3 cm), C3 (11.31–15.3 cm), and C4 (>15.3 cm).

Stage-specific projections further reinforce changes in demographic structure and projected population between regions. In Cuba, C4 plants remained the most abundant class throughout the observed period and were projected to decline gradually, while C1, C2, and C3 classes remained low and declined through the projection period (Figure 8). In Florida, projected totals decreased for all four classes, with particularly strong decreases in C1, C2, and C3. These projections were derived from aggregate annual class counts rather than individual plant histories, so again values should be interpreted as apparent class-level trajectories rather than direct estimates. Together, the observed and modeled results indicate declining *D. lindenii* populations in both Florida and Cuba, with the Florida population exhibiting a more rapid recent decline and stronger projected decrease.

## 3. Discussion

The long-term population and demographic data generated by these surveys provided clear outcomes for the three hypotheses evaluated in this study. Temporal population trends and regional differences showed that total count declined through time in both study regions, with a nearly four times greater rate of decline in Florida (8.8%) than Cuba (2.5%). Changes in demographic size structure also differed between regions, with smaller size classes declining in both populations while the number of plants in the largest size class increased within the Cuban population. Regional climate conditions and disturbance (temperature, precipitation, or tropical cyclone occurrence) were not associated with annual population growth.

### 3.1. Temporal Population Trends and Regional Differences

Although *D. lindenii* is considered one of the more intensively studied epiphytic orchids [35], conservation of the species remains uncertain. Previous work has addressed habitat requirements [7,22], insect pests [41,42], associated ants [43], floral fragrance [44], nectar chemistry [45], pollinators [14,15], mycorrhizal fungi [6,16], propagation [6], acclimatization [46], desiccation tolerance [47], and seedling establishment [32]. These studies have been motivated by observations of declining populations in southern Florida, a trend confirmed by our data. Previous monitoring studies in Cuba, paired with previous demographic modeling of *D. lindenii* of the Cuban populations, indicated a slowly declining population [48], which is consistent with the trend documented in our longer-term data set.

The greater rate of decline we observed in Florida would be better interpreted with expanded monitoring in other locations. Our study region on the FPNWR contains only a portion of the species remaining in the state. Substantial populations are also known to occur in neighboring protected areas, including Big Cypress National Preserve and Fakahatchee Strand State Preserve, with much smaller populations reported from Rookery Bay National Estuarine Research Reserve in Naples, FL, and Seminole Tribe of Florida Brighton Reservation in northeast Glades County, FL [9]. Continued standardized surveys across these areas will be important for determining whether the decline observed within the FPNWR is also occurring elsewhere in southern Florida.

Long-term population count studies remain uncommon for epiphytic orchids because individuals can be difficult to access and repeatedly monitor, and the studies that do exist exhibit substantial variation amongst species and years [4,25]. Einzmann et al. [5], for example, documented increasing number among many vascular epiphytes over a 21-year monitoring period in Panama. Here, however, the sustained declines observed for *D. lindenii* therefore appear to reflect specific stressors of these monitored populations rather than a universal trend for all orchids.

### 3.2. Changes in Dempgraphic Size Structure

One of the defining contrasts between Florida and Cuba is the trend in large reproducing (C4) plants. In Cuba, C1, C2, and C3 declined while C4 trend improved slightly (Table 1; Figure 8), such that at last monitoring, the C4 class represented 48% of the population in Cuba. Florida, in contrast, experienced losses of C1, C2, and C3 classes, but also the number of large C4 plants declined by four times as much during the monitoring period (at last monitoring 23% of Florida individuals were C4 size class). This distinction is particularly important because large established plants are not rapidly replaced and are the primary contributors to reproductive potential within populations. Given the declines in the smaller size classes, the already limited population of large reproducing individuals in Florida, which is being reduced by 1.6% annually, are unlikely to be soon replaced by smaller plants (Table 1).

The losses of large reproductive plants observed in this study may be further compounded by illegal collection. Orchid poaching, though rare, disproportionately targets large flowering individuals and may involve repeated removal from the same locations (Mike Owen, pers. comm.). More broadly, orchids are recognized as highly vulnerable to environmental and anthropogenic change [49], and specialist species such as *D. lindenii* that have co-habiting relationships cannot be easily returned once these relationships are disrupted [50]. Continued protection of the remaining reproductive individuals is therefore essential. The proposed protection of *D. lindenii* under the United States Endangered Species Act [51] may provide an additional mechanism for conserving remaining Florida populations.

The population declines observed in both locations were not accompanied by a reduction in flowering over time, with flower production fluctuating yearly in Florida and increasing in Cuba (Figure 5). Despite the availability of flowers, fruit production remained low in both regions. This may be due to a limitation of the primary pollinator of the species, the hawk moths (Lepidoptera: Sphingidae) [14]. This has been observed in other rare hawk moth-pollinated species such as *Platanthera leucophaea* [52] and may constrain successful replacement within these populations.

### 3.3. Regional Climate and Disturbance Association

Over half of all orchid species are now considered vulnerable to extinction throughout the world because of their sensitivity to acute environmental change [49]. *D. lindenii* depends on specific habitat conditions and co-habiting species. The species requires suitable host trees, compatible mycorrhizal fungi, and specific pollinators, each with their own potential response to alterations in climate and disturbance. Survival in this seasonally flooded habitat depends on adequate moisture, which is subject to climatic cycles and trends. Our study did not isolate the direct mechanism of population decline from annual surveys, but disruption of any of these components may affect population persistence.

Host-tree identity and spatial distribution may also influence the availability of suitable establishment sites. In a survey of 25 *D. lindenii* individuals in a Florida cypress strand, Pieterson et al. [22] documented 20 orchids (80%) on *F. caroliniana* and found nonrandom spatial clustering of both pop ash stems and ghost orchids. The study also recorded orchids on *Hippocratea volubilis* and *A. glabra*, underscoring that other woody hosts can occasionally support this orchid. In a different epiphytic plant community in a Mexican oak forest, Cortés-Anzúres et al. [53] showed that phorophyte identity, rather than host abundance alone, shaped epiphyte–host associations. Although this latter study did not examine *D. lindenii*, these findings suggest that the presence of trees alone is not necessarily an adequate measure of suitable orchid habitat. Local differences in host-tree distribution and microsite characteristics, including bark conditions, moisture availability, and compatible mycorrhizal fungi, could influence recruitment and persistence [6,16,22]. The contrast between host-tree associations in Florida and the broader range of documented hosts in Cuba further emphasizes the need to consider habitat and host availability at the population scale [7,35].

The regional climate analyses did not identify temperature or precipitation as statistical explanations for annual population changes in this study. However, the NASA POWER data indicates a mean temperature increase of approximately 1.47 °C in Florida and 1.05 °C in Cuba, which if trends continue may have a measurable impact on the species. Orchid populations in Florida are annually exposed to temperature extremes that do not occur in Cuba (Figure 3). Experimental work with *Lepanthes* demonstrated reduced photosynthetic performance and greater physiological stress under warmer and drier microclimatic conditions, indicating that epiphytic orchids can be sensitive to changes in temperature and moisture availability [54]. Environmental changes may have an impact on the orchids directly or may impact them indirectly via their co-habiting relationships. Greater and more persistent moisture may support fungal activity, particularly for the *D. lindenii* associate *Ceratobasidium* [6,16], as well as germination, and early seedling survival. Restoration of regional hydrology could therefore improve conditions for *D. lindenii* [28,55,56]. Therefore, the absence of persistent moisture may reduce the capability to meet replacement.

Tropical cyclones represent another potential climatic source of episodic disturbance throughout the range of *D. lindenii,* particularly when wind and precipitation activity damage host trees [26,27,29,30,31,32]. The present analyses did not detect an association between annual cyclone occurrence and annual population growth for either region. However, field observations and previous studies have documented canopy defoliation, host-tree damage, orchid mortality, and prolonged effects in response to major tropical cyclones on epiphytic orchids [26,27,31]. More research is needed to quantify factors such as local wind intensity, rainfall, canopy loss, altered humidity and light, and resultant host-tree mortality, or orchid demographic responses. This may have been completed had there been access to individual annual count responses. Continued monitoring following major storms will therefore remain important for detecting tropical cyclone impacts to *D. lindenii* populations.

We note the contrasting habitats of our study locations, Florida as seasonally flooded forests and Cuba as semi-deciduous forest offer an opportunity to compare potential climate-related stresses in different hydrological and geological settings [7]. From a regional standpoint, Florida has a more pronounced variation in mean maximum and minimum temperatures, whereas Cuba appears more stable (Figure 3). Direct measurements of climatic variables over the long term would provide a stronger basis for evaluating the climate-based mechanisms influencing *D. lindenii* populations in each region.

Host-tree availability, microhabitat conditions, and genetic variation may also influence the persistence of *D. lindenii*. Ecological niche modeling projects reduced climatic suitability under future warming scenarios [35], while historical logging and hydrological alteration have modified ghost orchid habitat in southern Florida [22]. Studies of other orchids have demonstrated how environmental conditions and genetic variation may influence population persistence. In *Platanthera integrilabia*, habitat structure was associated with differences in pollinator presence and interactions across study sites [57]. Similarly, research on *Bletia purpurea* in south Florida identified genetic differentiation and elevated inbreeding in some populations, highlighting the potential importance of habitat fragmentation and population connectivity [58]. However, genetic diversity does not always correspond with population growth, as demonstrated in a study of *Gymnadenia conopsea*, where population growth was associated with population size but not genetic diversity [59]. Our monitoring data cannot determine whether these factors contributed to the observed declines. Future research integrating genetic assessments, host-tree surveys, and local environmental measurements with long-term demographic monitoring would help clarify their relative importance.

### 3.4. Challenges of Restoration

The marked decline captured by our data, even for populations in protected areas such as the FNPWR, suggests that management options beyond habitat conservation be further explored as a means of promoting population persistence for this species. Artificial propagation and reintroduction may provide tools for supplementing declining populations.

Previous propagation research for *D. lindenii* has demonstrated that the species can be produced under laboratory conditions [6], and long-term outplanting work has shown that propagated seedlings can survive within natural Florida habitat [32]. A seven-year reintroduction study reported by Herdman et al. [32] found that seedling survival varied with microsite conditions, with greater survival when reintroduced near to mosses and lichens in contrast to other epiphytic species commonly found in the same habitat. The study also demonstrates why source material is important because the resulting established orchid population was not able to reproduce successfully.

The efficacy of orchid reintroduction has varied considerably among species and habitats but may prove a powerful tool for understanding the ecological complexities that constrain the survival of these specialist species. Restoration programs have produced successful initial establishment [32], but no study has yet documented a *D. lindenii* reintroduction that establishes a self-sustaining population. For orchids, reintroduction outcomes depend on site selection, habitat management, propagation methods, pollination, mycorrhizal associations [60]. In many cases, restoration is undertaken opportunistically when rare plant material becomes available, and without the advantage of research to support best practices. Many reintroduction outcomes are therefore variable and difficult to attribute cause [32,61].

Ex situ collections and reintroduction may help increase population size, but the sensitivity of large individuals identified here are not easily replaced. The reintroduction of *Cymbidium tracyanum* showed a substantially greater survival of larger seedlings than smaller seedlings, suggesting that plant size may influence establishment success in epiphytic orchids [62]. For most, however, it remains a substantial investment of time and resources to keep an orchid ex situ until it reaches a size that will confer the highest chance of establishment. Although the introduction of plants of any size will contribute to population augmentation, it could take many years to regenerate a population of established, large reproducing plants. In situ, *D. lindenii* can take nine years to reach reproductive maturity from seed (Mike Owen pers comm.). Restoration should therefore be viewed as a long-term compliment to the protection of existing populations rather than a replacement.

### 3.5. Study Limitations

Several limitations are important when interpreting these patterns derived from our data presented here. Regarding the climate and disturbance association analysis which used regional climate data, these broad landscape records do not represent the fine-scale environmental conditions experienced by individual orchids. Similarly, cyclone occurrence does not measure the local severity of disturbance. Additionally, the annual count data reported here limits the ability to detect nonlinear or delayed environmental responses. In addition, host-tree abundance and turnover, genetic variation and connectivity, and fine-scale environmental conditions were not quantified, preventing a direct evaluation of their contributions to the observed demographic trends.

Interpretation of the demographic classes also requires caution. These classifications describe plant size based on mean living root length rather than independently verified chronological age. Consequently, changes in class structure may reflect a combination of recruitment, growth, mortality, or changes in the living root systems of established individuals. Root mortality and replacement could alter the size-class assignment of a surviving plant without a corresponding change in its chronological age. Accordingly, the observed increase in Cuban C4 plants should be interpreted as a change in the number of the largest vegetative size class rather than as direct evidence of individual survival, aging, or transition from smaller classes. The demographic analyses are based on aggregate annual counts and size-class counts. Although individual plants were relocated annually and measured during surveys, the models used here do not estimate individual survival, growth, retrogression, recruitment, or fecundity transition probabilities. Changes among C1 through C4 therefore describe changes in observed size structure rather than direct individual transitions.

Finally, the regional comparison represents seven monitored sites within the FPNWR and the monitored population in Guanahacabibes National Park rather than contiguous populations throughout Florida and Cuba. The trajectories documented here should therefore not be interpreted as estimates for the species’ population within either region.

Despite these limitations, the long-term monitoring records reveal consistent conservation concerns. Florida is experiencing a more rapid decline, accompanied by losses of large plants and reduced flowering, while Cuba retains a substantial large plant component and greater flowering activity but continues to lose smaller size classes. Low fruit production in both regions further emphasizes that flowering alone does not ensure successful replacement. If recent demographic patterns persist, the modeled trajectories indicate continued declines in both regions. Conservation of *D. lindenii* will therefore require the protection of existing reproductive individuals, the maintenance of recruitment and establishment across the full demographic structure, the protection of suitable habitat, and the continued evaluation of restoration approaches.

## 4. Conclusions

The long-term declines documented for *D. lindenii* demonstrate why habitat conservation as a management strategy may not be sufficient to retain population persistence, and that coordinated conservation action is likely needed. The management challenges differ substantially between these regions. In Florida, the remaining populations occur within a previously contiguous ecological habitat that now spans a landscape divided among multiple management authorities, creating administrative boundaries that complicate coordination of conservation activities at a landscape scale. In Cuba, the remoteness of the monitored populations creates a different constraint, where access alone requires substantial logistical effort and limits the frequency of monitoring and potential management intervention.

The immediate priority should therefore be to protect the remaining wild populations while expanding standardized monitoring across the broader landscape. Comparable survey methods among Florida populations and continued monitoring in Cuba would allow managers to determine whether the trajectories documented here are occurring elsewhere and would provide the demographic baseline needed to evaluate future interventions. The next priority should be continued prospective research addressing population genetics, recruitment, reproductive success, seed viability, mycorrhizal associations, and controlled greenhouse and field experiments. These efforts should increasingly move from describing population decline toward identifying the processes that can be manipulated to improve recruitment and persistence.

Long-term conservation should build toward assisted restoration. Continued refinement of propagation methods, mycorrhizal inoculation, host tree and microsite selection, reintroduction, and population augmentation will be necessary if depleted demographic components are to be restored. These actions should be accompanied by continued monitoring after outplanting so that survival, establishment, and reproduction can be evaluated and management adjusted accordingly. Previous propagation and reintroduction research demonstrates that such restoration is possible, but also that success depends strongly on microsite conditions and the continued refinement of methods.

The broader value of conserving the ghost orchid extends beyond this species. Many imperiled epiphytic plants lack the public visibility, long-term datasets, and research investment that have accumulated around *D. lindenii*. The conservation framework being developed through this research can therefore provide a model for approaching other rare epiphytes for which managers currently lack comparable information. Establishing a sequence that begins with standardized monitoring, advances through targeted demographic and biological research, and ultimately supports evidence-based restoration can make the scope and cost of conservation more predictable and achievable. Continued collaboration among researchers, land managers, botanical institutions, conservation organizations, and local partners will be essential not only for the recovery of *D. lindenii*, but also for translating the lessons learned from this species into broader strategies for conserving rare epiphytic plants.

These challenges are representative of a broader problem in rare epiphyte conservation. Land managers are generally responsible for entire landscapes and biological communities, while the detailed demographic, reproductive, fungal, and environmental information needed to manage individual rare species are often unavailable or prohibitively costly to obtain. *Dendrophylax lindenii* is unusual because sustained public interest, scientific attention, and collaboration have produced a comparatively detailed understanding of its biology. Research has now addressed population demography, pollination, mycorrhizal associations, propagation, seedling establishment, and habitat requirements, providing a foundation from which more active restoration can realistically be considered.

## 5. Materials and Methods

### 5.1. Study Sites

Two study locations, one in the United States (FPNWR) and the other in Cuba (Guanahacabibes National Park), were chosen for long-term surveys due to the large number of *D. lindenii* known to occur in each and their inaccessibility to humans. The FPNWR is uniquely situated because it includes the northern portion of the Fakahatchee Strand in Collier County where a high concentration and diversity of North America’s epiphytic orchids reside within a narrow (2 × 35 km) strip with standing water. Within the 10,684 ha FPNWR, 27 orchid species in 17 genera have been documented [63], including ca. one-quarter of all *D. lindenii* thought to occur naturally in Florida. Most of the epiphytes including *D. lindenii* are confined to strand swamps and sloughs shaded by a canopy of bald cypress (*Taxodium distichum*, Cupressaceae) on branches of smaller understory trees, namely pop ash (*Fraxinus caroliniana*, Oleaceae) and pond apple (*Annona glabra*, Annonaceae), primarily the former [7,32]. Within the FPNWR, *D. lindenii* occurs in seven distinct populations, the largest of which lies within the wettest location.

**Figure 9:**
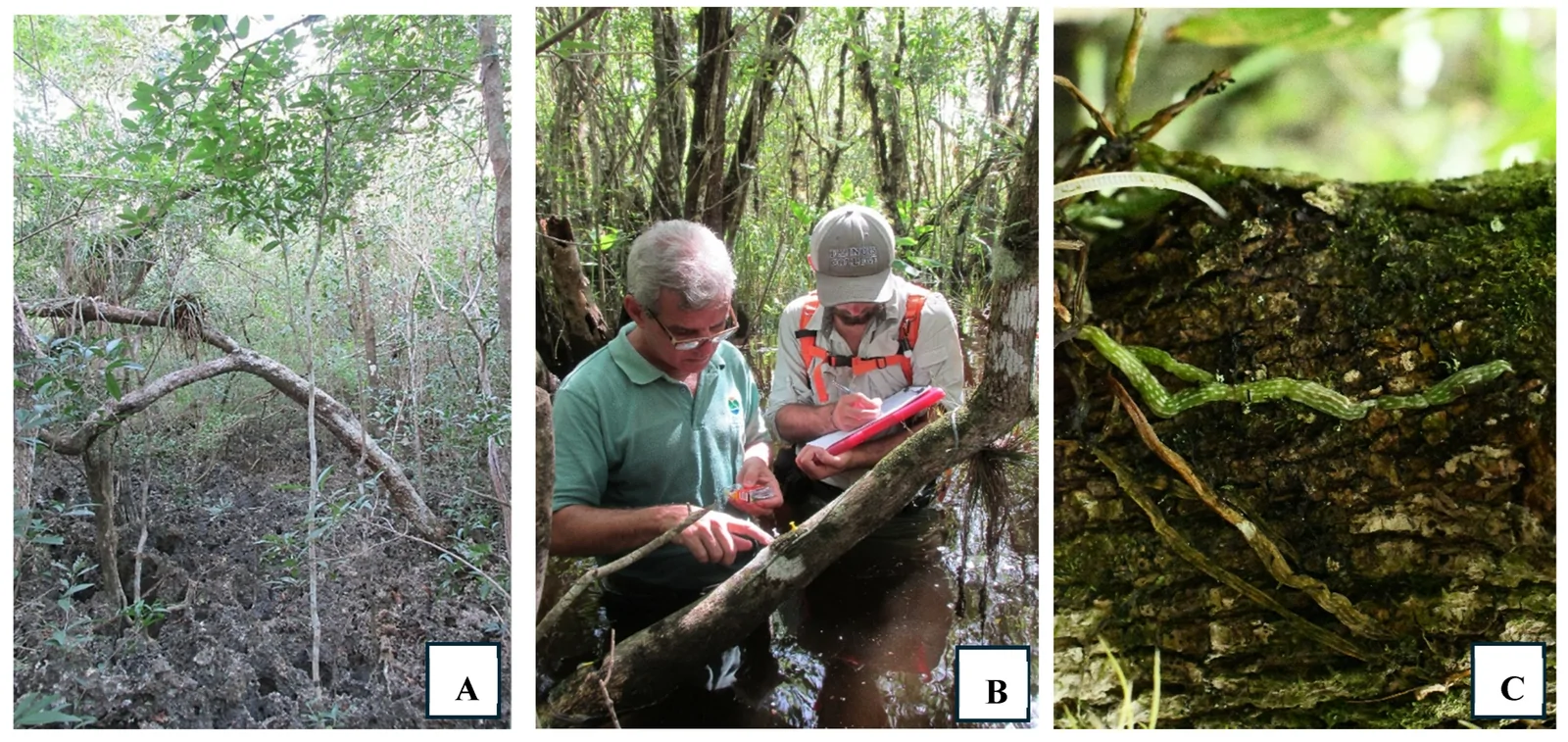
Habitats and root morphology of *D. lindenii* in Cuba and Florida. (**A**) Semi-deciduous forest habitat in Guanahacabibes National Park, Cuba, showing jagged reef limestone beneath host trees and the absence of standing water. Photograph: L.W. Zettler. (**B**) Flooded forest habitat within the FPNWR, showing *D. lindenii* seedlings on the bark of *Fraxinus caroliniana*. Photograph: L.W. Zettler. (**C**) Living gray-green roots of *D. lindenii* attached to host-tree bark, showing the characteristic linear white pneumathodes used to identify the species during field surveys. Photograph: A.R. Herdman.

In Cuba, surveys were carried out in Guanahacabibes National Park located on the westernmost tip of the island in the Guanahacabibes Peninsula Biosphere Reserve in the Pinar del Río Province. This location contains *D. lindenii* and 31 other orchid species (three endemics), many of which are epiphytes attached to trees rooted on karst limestone devoid of standing water [7,9] (Figure 9A). Nearly all *D. lindenii* in Cuba are thought to be confined to the Guanahacabibes Peninsula, all of which exist there as epiphytes on at least 18 host-tree species [7]. Other details that fully characterize the two study sites are reported in Mujica et al. [7]. The precise locations of monitored populations are intentionally withheld to minimize the risk of illegal collection and disturbance to this rare and conservation-sensitive species.

### 5.2. Field Survey Protocol

Field surveys were carried out annually during the growing season in both locations during the summer months following the protocols reported in Müjica et al. [28]. In the FPNWR, this took place during the month of July starting in 2015 and ending in 2024. In Cuba, surveys began a decade earlier (2006).

**Figure 10:**
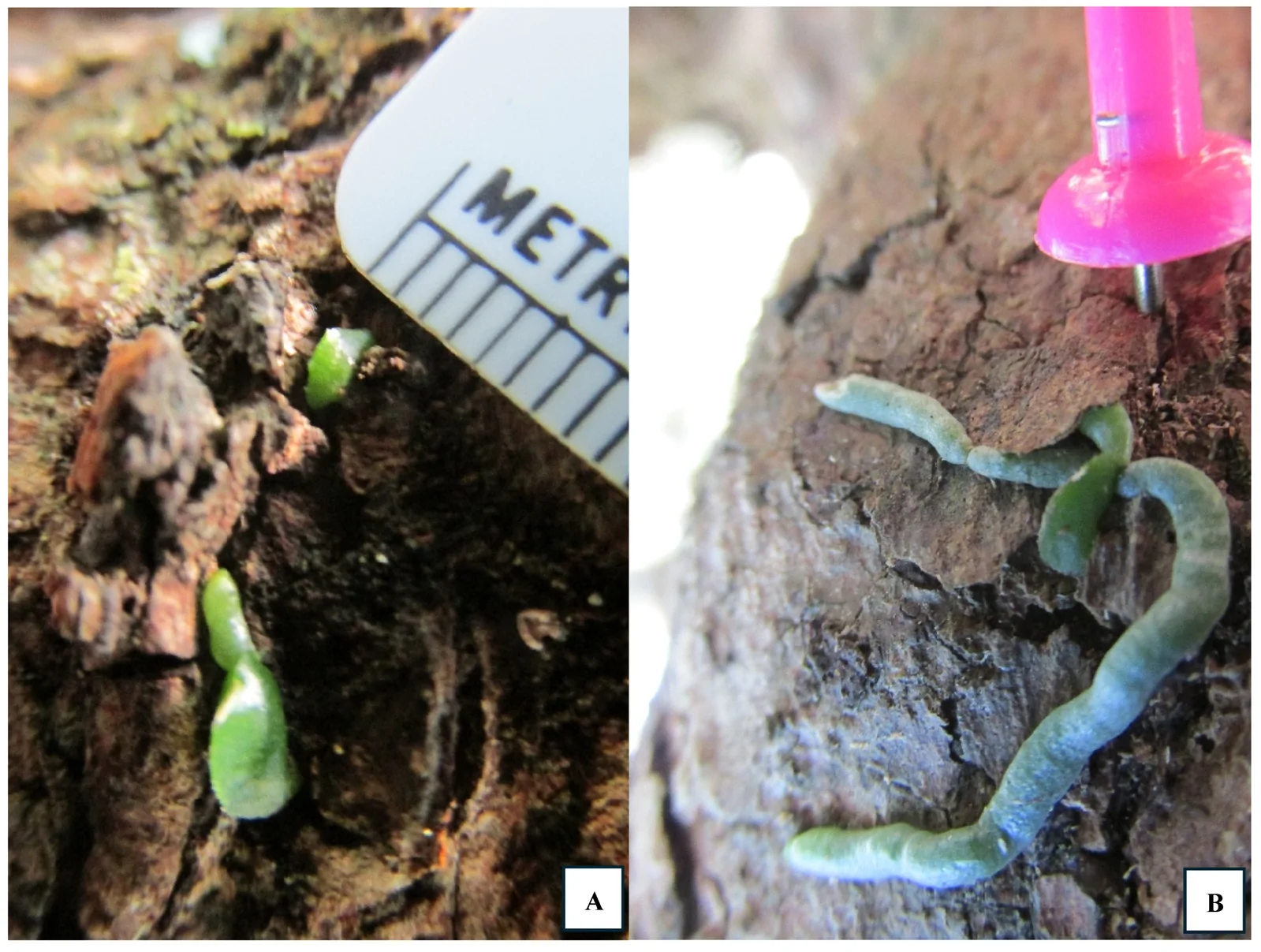
Early seedling development of *D. lindenii* on the bark of *Erythroxylum aerolatum* in Guanahacabibes National Park, Cuba. (**A**) Two young seedlings photographed in January 2016, showing the dorsal crest preceding root development. The lower seedling was estimated to have germinated approximately three months before the photograph, based on the development of its first root. Each ruler increment represents 1 mm. (**B**) A seedling photographed on the same host tree in June 2016, showing three gray-green roots emerging from the dorsal crest. The dorsal crest has been hypothesized to contribute to water storage during early development and may eventually detach as the roots become established on the bark surface. Scale bar = 0.5 cm. Photographs: L.W. Zettler.

In both study sites (Florida, Cuba), most *D. lindenii* were positioned on the main trunk low enough (<3 m) to be observed and measured directly without the need for special climbing equipment (Figure 9B). Orchids on branches higher in the canopy were inspected visually. Special attention was given to examining bark surfaces for the presence of small-stage seedlings which were distinguished by the presence of small linear white flecks (pneumathodes) on root surfaces (Figure 9C) that are absent on the roots of two other leafless orchid species (*Campylocentrum pachyrrhizum*, *Dendrophylax porrectus*). Because *D. lindenii* is entirely leafless, once roots begin to grow outward as early seedlings, plant size was defined by the length of the living roots present and served as the basis for our stated variable in the analysis (Figure 10). Roots of larger individuals were traced to their point of origin, making accurate counting possible. Orchids that occurred in clusters were individually marked using detailed distribution data at their point of discovery. Root growth data were limited to individuals measured the previous year, not newly discovered orchids. Reproductive output was quantified from annual counts of inflorescences and fruits recorded during each survey.

Population size was assessed using annual counts of living *D. lindenii* individuals recorded within established monitoring areas at each study location. Individual orchids were documented in association with their respective host trees, which were geographically referenced and marked to facilitate repeated monitoring. Surveys included the assessment of previously documented individuals and the identification of newly encountered plants. In Florida, monitoring was conducted within seven sites on the FPNWR while Cuban surveys encompassed the only population in Guanahacabibes National Park. These populations were monitored annually to document temporal changes in count, demographic size structure, and reproductive output.

For the classification of *D. lindenii* plants we included only living roots, their mean lengths, and their individual lengths. In cases where an orchid’s root mass could not be accurately quantified, for example, because of restricted access due to height, that individual was recorded as an outlier for root measurement. Plants were assigned to four demographic size classes based on mean living root length. These classes represent increasing vegetative size rather than chronological age. Because *D. lindenii* is leafless and vegetative development is primarily through its root system, mean living root length provides a biologically relevant measure of plant size. Class 1 (C1) included individuals with a mean root length of 0 to 8.0 cm, Class 2 (C2) included individuals with a mean root length of 8.1 to 11.3 cm, Class 3 (C3) included individuals with a mean root length of 11.31 to 15.3 cm, and Class 4 (C4) included individuals with a mean root length greater than or equal to 15.3 cm. Individual plants may change size class as living roots grow, senesce, or are replaced, so the classes should not be interpreted as fixed age categories or sequential developmental stages.

Reproductive maturity was evaluated separately from vegetative size. Plants with a current inflorescence, or evidence of an inflorescence from the previous year, were considered reproductively mature. Thus, reproductive maturity was not assigned solely on the basis of membership in C4. After sampling, the previous year’s inflorescence was removed to ensure accuracy the following year.

### 5.3. Demographic Trends, Population Trajectories, and Environmental Associations

Modeling demographics were based on the same protocol described in Mujica et al. [28]. Size-structured IPMs were constructed for each yearly transition (e.g., 2020–2021, 2021–2022) to estimate temporal variability in demographic rates. This was accomplished by grouping year-specific survival, growth and reproductive rates into four separate matrices consisting of 45 size categories (1 cm width each). These matrices were then analyzed as a time-invariant model. Demographic analyses were based on annual counts of total living individuals and annual counts assigned to C1, C2, C3, and C4. Analyses were conducted separately for Florida and Cuba using the available monitoring periods of 2015 to 2024 and 2006 to 2023, respectively.

Simplified annual finite rates of population change were calculated for each region as λ = N_t+1_/N_t_, where N_t_ represents the total class-structured count in year t and N_t+1_ represents total class-structured count in the following year. An overall λ was calculated as the geometric mean of annual λ values across the study period. These estimates were used to describe changes in the observed population total and to compare broad population trends between Florida and Cuba. Uncertainty in overall λ was estimated using 5000 nonparametric bootstrap resamples of annual log-transformed λ values, from which 95% confidence intervals were calculated.

Long-term trends in total live count were evaluated separately for Florida and Cuba using Mann–Kendall tests and Theil–Sen slopes. Log-linear regressions of total plants against year were additionally used to estimate annual proportional rates of change and 95% confidence intervals. To test whether the rates of decline differed between regions, a population × year interaction was evaluated using years for which count data were available for both populations.

Temporal trends in the number of plants in each demographic size class were evaluated separately for C1, C2, C3, and C4 within each region using the same Mann–Kendall and Theil–Sen framework applied to total count. Mann–Kendall tests evaluated evidence for temporal change, while Theil–Sen slopes quantified the median change in the number of individuals per year. Because four class-specific trend tests were conducted within each population, *p* values were adjusted using the Benjamini–Hochberg procedure to control the false discovery rate. Statistical significance of class-specific trends was evaluated using the adjusted *p* values.

Log-linear regressions of annual class count against year were also fitted separately for each size class. Regression coefficients were exponentiated to estimate the average annual proportional change in number of plants, and 95% confidence intervals were calculated from the corresponding regression coefficient confidence limits. To distinguish changes in absolute class count data from changes in demographic composition, the proportional contribution of each class was calculated annually as the number of individuals in that class divided by the sum of C1 through C4 individuals recorded in the same year. Temporal trends in these proportions were evaluated using Mann–Kendall tests and Theil–Sen slopes, with slopes expressed as percentage points per year. Benjamini–Hochberg adjustment was again applied across the four class tests within each population. These analyses allowed a class to be evaluated separately for changes in absolute total and changes in its relative contribution to the class-structured population.

To evaluate simplified stage-based dynamics, we fit a four-stage projection using annual counts of C1, C2, C3, and C4 plants. For each region, a non-negative transition matrix was estimated to best predict the observed class structure in year t + 1 from the class structure observed in year t. The resulting matrices were then used to project class-specific population trajectories forward and compare modeled values with observed class counts. Uncertainty in the dominant eigenvalue was evaluated by bootstrap resampling of annual transition pairs.

Available data consisted of aggregate annual class counts rather than individual plant histories; matrix elements were interpreted as apparent class-level contributions rather than true estimates of individual survival, growth, retrogression, or fecundity. Therefore, these models were used as simplified descriptors of population trajectory and class-structure change, rather than as full individual-based demographic models. The class-specific Mann–Kendall and Theil–Sen analyses described above likewise quantify temporal changes in observed annual class count and composition rather than individual transitions among classes.

Daily climate data for the FPNWR and Guanahacabibes National Park were obtained from the NASA Prediction of Worldwide Energy Resources (POWER) project for 2005–2024. Variables included daily minimum (T2M_MIN), mean (T2M), and maximum (T2M_MAX) 2 m air temperature (°C) and corrected precipitation (PRECTOTCORR; mm day^−1^). Daily values were summarized to monthly and annual scales. Temporal trends in monthly temperature and precipitation were evaluated separately for each region using generalized additive models that included a smooth effect of time and a cyclic monthly term to account for seasonality.

Relationships between climate and population dynamics were evaluated by relating annual climate summaries to annual log(λ). Annual minimum, mean, and maximum temperature, temperature extremes, total precipitation, and wet- and dry-season precipitation were evaluated individually using linear regressions and Spearman’s rank correlations. Same-year and one-year-lagged associations were examined, with Benjamini–Hochberg adjustment applied to multiple climate tests.

To evaluate temporal correspondence with tropical cyclone disturbance, annual population transitions were classified according to whether a named tropical cyclone occurred during the corresponding year. Mean log(λ) was compared between hurricane-exposed and non-hurricane intervals using bootstrap confidence intervals and permutation tests. This analysis evaluated the timing of hurricane occurrence relative to annual population change and did not quantify the persistent effects of canopy loss, host-tree damage, altered microclimate, or other delayed ecosystem responses.

POWER meteorological products were obtained from the NASA Prediction Of Worldwide Energy Resources (POWER) Project (NASA Langley Research Center, Hampton, VA, USA) and are derived from NASA’s Modern-Era Retrospective Analysis for Research and Applications, Version 2 (MERRA-2). All data processing, statistical analyses, demographic modeling, and figure generation were conducted in R version 4.5.1 (R Foundation for Statistical Computing, Vienna, Austria) [64].

## Figures and Tables

**Table 1:** Temporal trends in total number of plants and demographic size classes of *D. lindenii* in Florida and Cuba. Categories represent four size classes based on mean living root length: C1 (0–8.0 cm), C2 (8.1–11.3 cm), C3 (11.31–15.3 cm), and C4 (>15.3 cm). Mann–Kendall tests evaluated temporal trends. Theil–Sen slopes estimate the median change in the number of individuals per year. Benjamini–Hochberg adjusted *p* values account for the four simultaneous class tests conducted within each population. Annual proportional change and 95% confidence intervals are estimated using log-linear regression.

Category	Kendall τ	Mann–Kendall *p*	BH Adjusted *p*	Theil–Sen Slope, yr^−1^	Annual Change yr^−1^ (95% CI)
Florida (2015–2024)					
Overall	−0.822	0.0013	NA	−35.7	−8.8 (−11.4 to −6.1)
C1	−0.644	0.0123	0.0245	−16.5	−11.5 (−16.4 to −6.4)
C2	−0.386	0.1491	0.1988	−3.0	−8.1 (−14.8 to −0.9)
C3	−0.764	0.0030	0.0122	−12.0	−16.7 (−23.6 to −9.1)
C4	−0.333	0.2105	0.2105	−4.0	−1.6 (−6.5 to 3.5)
Cuba (2006–2023)					
Overall	−0.761	<0.001	NA	−6.36	−2.5 (−3.2 to −1.8)
C1	−0.589	0.0008	0.0008	−2.9	−5.6 (−8.3 to −2.9)
C2	−0.779	<0.0001	<0.0001	−3.5	−10.6 (−12.9 to −8.3)
C3	−0.713	<0.0001	0.0001	−2.3	−6.5 (−8.4 to −4.7)
C4	+0.625	0.0004	0.0005	+2.25	+2.4 (+1.3 to +3.4)

## Data Availability

The data presented in this study are available on request from the corresponding author. The data are not publicly available due to the rarity of the species investigated and to limit the access to location data.
